# Characteristics and outcomes of out-of-hospital cardiac arrest patients before and during the COVID-19 pandemic in Thailand

**DOI:** 10.1186/s12245-022-00444-2

**Published:** 2022-09-09

**Authors:** Phatthranit Phattharapornjaroen, Waratchaya Nimnuan, Pitsucha Sanguanwit, Pongsakorn Atiksawedparit, Malivan Phontabtim, Yahya Mankong

**Affiliations:** 1 Department of Emergency Medicine, Faculty of Medicine, Ramathibodi Hospital, Mahidol University, Bangkok, 10400 Thailand; 2 Chakri Naruebodindra Medical Institute, Faculty of Medicine, Ramathibodi Hospital, Mahidol University, Samut Prakan, 10540 Thailand

**Keywords:** Out-of-hospital cardiac arrest, COVID-19, Pandemic, SARS-CoV-2, Emergency department, Return of spontaneous circulation, Survival to admission, 30-day survival

## Abstract

**Background:**

Out-of-hospital cardiac arrest (OHCA) remains one of the leading causes of death worldwide, and bystander CPR with public-access defibrillation improves OHCA survival outcomes. The COVID-19 pandemic has posed many challenges for emergency medical services (EMS), including the suggestion of compression-only resuscitation and recommendations for complete personal protective equipment, which have created operational difficulties and prolonged response time. However, the risk factors affecting OHCA outcomes during the pandemic are poorly defined. This study aimed to assess the characteristics and outcomes of OHCA patients before and during the COVID-19 pandemic in Thailand.

**Methods:**

This single-center, retrospective cohort study used data from electronic medical records and EMS paper records. All OHCA patients who visited Ramathibodi Hospital’s emergency department before COVID-19 (March 2018 to December 2019) and during COVID-19 (March 2020-December 2021) were identified, and the number of emergency department returns of spontaneous circulation (ED-ROSC) and characteristics in OHCA patients before and during the COVID-19 pandemic in Thailand were collected.

**Results:**

A total of 136 patients were included (78 men [59.1%]; mean [SD] age, 67.9 [18] years); 60 of these were during the COVID-19 period (beginning March 2020), and 76 were before the COVID-19 period. The overall baseline characteristics that differed significantly between the two groups were bystander witness and mode of chest compression (*p*-values < 0.001 and < 0.001, respectively). The ED ROSC during the COVID-19 period was significantly lower than before the COVID-19 period (26.67% vs. 46.05%, adjusted OR 0.21, *p*-value < 0.001). There were significant differences in survival to admission between the COVID-19 period and before (25.00% and 40.79%, adjusted OR 0.26, *p*-value 0.005). However, 30-day survivals were not significantly different (3.3% during the COVID-19 period and 10.53% before the COVID-19 period).

**Conclusions:**

During the COVID-19 pandemic in Thailand, ED ROSC and survival to admission in out-of-hospital cardiac arrest patients were significantly reduced. Additionally, the witness responses and mode of chest compression were very different between the two groups.

**Trial registration:**

This trial was retrospectively registered on 7 December 2021 in the Thai Clinical Trial Registry, identification number TCTR20211207006.

**Supplementary Information:**

The online version contains supplementary material available at 10.1186/s12245-022-00444-2.

## Introduction

Out-of-hospital cardiac arrest (OHCA) has remained one of the leading causes of death worldwide for decades (1–3). Several studies reported the influences of prehospital variables on OHCA outcomes like survival to discharge and one-year survival rate; those influences are bystander CPR, witnessed arrest, and Automated External Defibrillator (AED) (4–8). However, there have been some barriers to performing chest compression before first medical contact. One of the factors leading to a low rate of bystander CPR is a shortage of knowledge about the basic life support (5,9). Other barriers include fear of infectious disease, worries about mouth-to-mouth ventilation, and a high level of stress during the occurrence (10). The Pan Asia Resuscitation Outcomes Study (PAROS), a study of OHCA characteristics in Asian countries, reported that the survival-to-discharge rate varied from 0.5 to 8.5%, and survival with good neurological function ranged from 1.6% to 3% (11). These outcomes are notably low in comparison to results reported in studies in European countries and the United States (3); this is presumably secondary to ineffective or delayed chest compression either from bystander witnesses or prehospital medical teams because the rate of successful ROSC decreases by 7%–10% for every minute waited (12). Therefore, to minimize mortality and convey patients with preferable neurological outcomes, emergency medical service (EMS) performance of cardiopulmonary resuscitation (CPR) is considered the key element to delivering high-quality procedures in the shortest response time (13,14). The study showed a survival rate increased by 27.1% if EMS is present within 2 min (15).

The recent outbreak of novel coronavirus disease-2019 (COVID-19) has provided challenges for EMS, especially in managing time-dependent response cases such as OHCA (16–19). These challenges have affected prehospital ROSC and survival to admission for OHCA patients. The latest OHCA recommendations from the American Heart Association (AHA) suggest compression-only resuscitation and public-access defibrillation, that healthcare professionals should use personal protective equipment for aerosol-generating procedures during resuscitation, and that healthcare providers should consider defibrillation before donning aerosol-generating personal protective equipment in situations where the provider assesses the benefits may exceed the risks (20). Many countries adopted the guidelines and changed their management (18,21,22). As a result, the pandemic period was associated with lower survival to admission in several studies (23,24). Moreover, recent studies have shown an upward trend in adult OHCA in Singapore from 26.2 in 2019 to 28.8 in 2020 per 100,000 population. In Paris, weekly incidences increased from 13.42 to 26.64 per million inhabitants (24,25).

Prehospital care in Thailand has been operating for over two decades. The service follows a model that is a mix of the Anglo-American and Franco-German emergency medical systems (26). During the COVID-19 pandemic, Thailand has faced a large volume of infectious cases that occupied the major hospital and prehospital resources, with a report of an increased mortality rate (27). Thai health care services responded to the pandemic by modifying critical parts of guidelines according to international recommendations (28). However, little is known about OHCA, one of the leading causes of prehospital mortality, especially during the pandemic; we hypothesized that the survival rate would fall because of various protocol modifications that could delay procedures. Thus, we aimed to assess the characteristics of OHCA patients and their outcomes in terms of return of spontaneous circulation (ROSC) at the emergency department (ED), survival to admission, 30-day survival, and good cerebral performance category (CPC); Category 1–2 before and after the COVID-19 pandemic in Thailand.

## Methods

### Study design and setting

We performed a single-center, retrospective cohort study using data from electronic medical records (EMR) and EMS paper records of Ramathibodi Hospital, a tertiary care university hospital in Bangkok that employs professionals in various medical professions and utilizes advanced-technology facilities to treat complicated cases. The study collected data from all OHCA patients who visited Ramathibodi Hospital’s emergency department from March 2018 to December 2019; before the COVID-19 period, and from March 2020 to December 2021; during the COVID-19 period. All ED visits and patients’ data were digitally recorded and stored on a secure system that ensured privacy. Informed consent was waived as the data were retrospectively collected and were anonymous. This study was approved by The Committee on Human Rights Related to Research, Faculty of Medicine, Ramathibodi Hospital, Mahidol University (COA. MURA2020/997).

### EMS setting

In Thailand, the EMS services are hospital-based ambulances with independent central dispatch centers. The dispatch centers receive calls from throughout the regions and dispatch ambulances to patients based on their locations. Within the Ramathibodi Hospital area, there are approximately 600,000 population (29), and one university hospital performs as a center for receiving all information from the central dispatcher. After that, three university hospitals, private hospitals, and advance ambulances by a volunteer in the area are dispatched on a rotational basis depending on availabilities and frequency by the university hospital. The team leader for the university ambulances is a doctor in emergency training, yet the leaders for other ambulances are either nurses or paramedics. Patients are admitted to each ED based on their home registration or preferences or from the area of the hospital’s responsibilities. Nonetheless, in other cases, families or patients have the chance to decline their health care rights and request a hospital of their choice. The government supports the EMS system, while direct calls to the private hospital are self-paid.

### Study Participants

**Fig. 1:**
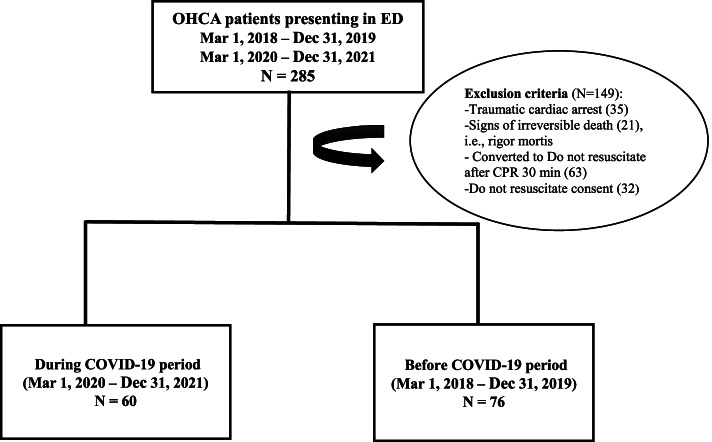
illustrates the study protocol

The OHCA cases were identified using the international classification of disease 10^th^ (ICD-10); records diagnosed with ‘cardiac arrest’ or tagged with ‘death before arrival’ was reviewed for inclusion and exclusion criteria. All OHCA patients 18 years of age or older who were brought to the emergency room at Ramathibodi Hospital by any mode of transportation were included in the study. Exclusion criteria were traumatic out-of-hospital cardiac arrest, evidence of irreversible death (e.g., rigor mortis, dependent lividity), EMS-treated patients whose CPR was initiated for a short period of time but converted to Do Not Resuscitation (DNR), and patients who had a valid do-not-resuscitate order. Although mid-February 2020 was the period of first case identification, the rapid spread of the disease began on March 1^st^, 2020, in the capital city, Bangkok. Thus, the During COVID-19 period group was defined as patients who had visited between March 1, 2020, and December 31, 2021. The comparison group (before the COVID-19 period) was defined as patients who had been seen between March 1, 2018, and December 31, 2019. The study flow chart had demonstrated in Fig. 1**,** and we performed the subgroup analysis on patients admitted to the emergency department by Ambulance (who used the hospital dispatch system).

### Data collection

We collected patient characteristics including age, gender, comorbidities, location of cardiac arrest, mode of transportation, EMS response time, whether the event was bystander-witnessed, whether bystander CPR was attempted, initial rhythm, etiology of cardiac arrest, and resuscitative intervention. The primary outcome was ED ROSC which is referred to as sustained ROSC at ED (30). Moreover, survival to admission, 30-day survival, and 30-day good cerebral performance category (CPC) scores were collected and calculated as secondary outcomes.

*OHCA,* out-of-hospital cardiac arrest; *ED,* emergency department; *EMS*, emergency medical services; *ROSC,* return of spontaneous circulation.

### Sample size

The sample size calculation was based on the outcome of ED ROSC of 7.8% during the COVID-19 period and 28.2% before the COVID-19 period (31). The probabilities of type I error (α = 0.05) and type II error (β = 0.20) were estimated using the formula, and the allocation ratio (N2/N1) was 1.0. The total sample size required was 110 patients, divided into 55 patients from the COVID-19 period and 55 from the comparison period (before COVID-19).

### Statistical analysis

Descriptive statistics were calculated for all clinical characteristics and relevant variables; continuous variable data were calculated using an independent t-test or Mann–Whitney U test and are presented as means (standard deviations; SD) for variables that are normally distributed or medians in non-parametric tests. Categorical data were calculated using a chi-square test or Fisher’s exact test, as appropriate, and are presented as percentages. The outcomes analysis of ED ROSC, survival to admission, 30-day survival, and 30-day CPC score were compared using multivariable logistic regression analysis for binary outcomes. Additionally, the generalized linear regression with a log link and Gaussian distribution robust variance estimation to analyze risk difference. We reviewed the literature for the covariates which affected the outcomes (11,32–35). The outcomes were adjusted for the following variables: age, comorbidities, cardiac etiology, public location, bystander CPR, EMS response, initial shockable rhythm, and mode of chest compression. The OHCA patients admitted to the ED by ambulances were specifically analyzed as subgroup analysis (hospital dispatch subgroup) to evaluate the differences in their outcomes before and after COVID-19 periods.

All tests were two-sided, and values were considered statistically significant with a p-value < 0.05. We performed all data analysis using Stata version 16 (Stata Corp LLC, College Station, TX, USA).

## Results

In this study, 285 OHCA patients were included in the emergency department’s electronic medical records from March 1, 2018, to December 31, 2019, and March 1, 2020, to December 31, 2021, of whom 149 patients were excluded because of traumatic OHCA, irreversible death, converted to Do Not Resuscitation, do-not-resuscitate order, or missing data. The remaining 136 patients met the eligibility criteria; 60 were allocated to the COVID-19 period group and 76 to the COVID-19 period group.

Overall baseline characteristics are presented in Table 1. The mean age was 67.98 years; 59.09% of patients were male. Patients in the COVID-19 period had significantly fewer bystanders witness lower than before the COVID-19 period (81.67% vs. 100.00%, *p* < 0.001, respectively). In addition, mechanical chest compression was significantly more used in the COVID-19 period than before the COVID-19 period (93.33% vs. 57.89%, *p* < 0.001). There were no statistically significant differences between the two groups in terms of comorbidities, etiology of cardiac arrest, location of occurrence of the OHCA, bystander-CPR, EMS response time, number of patients who received initial shockable rhythm, defibrillation, or endotracheal intubation.

**Table 1:** Baseline characteristics of out-of-hospital cardiac arrest patients During the COVID-19 period and Before the COVID-19 period

**Baseline characteristics**	**All** **(*N* = 136)**	**During COVID-19 period** **(*N* = 60)**	**Before COVID-19 period** **(*N* = 76)**	***p*-value**
Age, years	67.98 ± 18.43	65.42 ± 19.43	70.00 ± 17.48	0.15
Male sex, N (%)	78 (59.09)	33 (55.00)	46 (60.53)	0.60
Comorbidity status, N (%)	87 (63.97)	33 (55.00)	54 (71.05)	0.07
Hypertension	67 (49.26)	28 (46.67)	39 (51.32)	0.61
Diabetes mellitus	36 (26.47)	15 (25.00)	21 (27.63)	0.85
Dyslipidemia	35 (25.74)	14 (23.33)	21 (27.63)	0.69
Chronic kidney disease	19 (13.97)	7 (11.67)	12 (15.79)	0.62
Ischemic heart disease	20 (14.71)	7 (11.67)	13 (17.11)	0.47
Cerebrovascular disease	19 (13.97)	7 (11.67)	12 (15.79)	0.62
Asthma/COPD	9 (6.62)	3 (5.00)	6 (7.89)	0.73
Cardiac etiology, N (%)	43 (31.62)	18 (30.00)	25 (32.89)	0.85
Public location, N (%)	64 (47.06)	26 (43.33)	38 (50.00)	0.49
Bystander-witnessed, N (%)	125 (91.91)	49 (81.67)	76 (100.00)	< 0.001
Bystander CPR, N (%)	54 (39.71)	28 (46.67)	26 (34.21)	0.16
EMS transportation, N (%) -Advanced life support (ALS) -Basic life support (BLS)	66 (48.53) 63 (46.32) 3 (2.21)	33 (55.00) 32 (53.33) 1 (1.67)	33 (43.42) 31 (40.79) 2 (2.63)	0.23 0.17 1.00
EMS response time, minutes (mean ± SD)	11.87 ± 5.74 (*n* = 39)	12.33 ± 5.74 (*n* = 24)	11.13 ± 3.85 (*n* = 15)	0.48
Initial shockable rhythm, N (%)	79 (58.09)	35 (58.33)	44 (55.89)	1.00
Mode of compression, N (%)				< 0.001
-Mechanical devices -Manual	100(73.53) 36(26.47)	56 (93.33) 4(6.67)	44 (57.89) 32(42.11)	
Defibrillation, N (%)	34 (25.00)	19 (31.67)	15 (19.74)	0.12
Endotracheal intubation, N (%)	128 (94.12)	55 (91.67)	73 (96.05)	0.30
Coronary reperfusion, N (%)	7 (5.15)	3 (5.00)	4(5.26)	1.00
Targeted temperature management, N (%)	4 (2.94)	0(0)	4(5.26)	0.13

*COPD,* chronic obstructive pulmonary disease; *EMS,* emergency medical service

The univariable and Multivariable analysis factors of the outcome; ED ROSC, before the COVID-19 period and during the COVID-19 period are shown in Table 2. The univariable and Multivariable analysis factors of other secondary outcomes before and during the COVID-19 period are shown in supplement 1, 2 and 3.

**Table 2:** Univariable and Multivariable analysis factors of ED ROSC during the COVID-19 period and before the COVID-19 period

Variable	Univariable analysis				Multivariable analysis			
	**Odd ratio**	**95%CI**	**P value**	**Coef**	**Odd ratio**	**95%CI**	**P value**	**Coef**
Covid period	0.43	0.20–0.88	0.02	-0.85	0.21	0.08 -0.53	< 0.001	-1.56
Age	0.98	0.97–1.00	0.13	-0.01	0.97	0.95—0.99	0.03	-0.03
DM	1.74	0.80–3.77	0.16	0.55	2.34	0.79—6.94	0.12	0.85
HT	0.87	0.43–1.74	0.69	-0.14	0.91	0.31—2.66	0.86	-0.10
Dyslipidemia	1.35	0.62–1.96	0.45	0.30	1.68	0.49—5.72	0.41	0.52
Chronic kidney disease	0.97	0.35–1.64	0.95	-0.03	0.76	0.21—2.70	0.67	-0.28
Heart disease	0.68	0.24–1.89	0.46	-0.39	0.58	0.18—1.92	0.37	-0.54
Cerebrovascular disease	0.97	0.35–2.64	0.95	-0.03	1.38	0.43—4.43	0.59	0.32
Chronic lung disease	2.20	0.56–8.60	0.26	0.79	1.41	0.27—7.39	0.69	0.34
Cardiac etiology	0.54	0.24–1.17	0.12	-0.62	0.48	0.18—1.28	0.14	-0.73
Public location	1.66	0.82–3.33	0.16	0.51	1.34	0.58—3.11	0.50	0.29
Bystander CPR	1.10	0.54–2.24	0.79	0.10	7.43	1.25—44.14	0.03	2.01
EMS transport Mode	0.71	0.35–1.42	0.33	-0.35	0.14	0.02 – 0.95	0.05	-1.94
Initial shockable rhythm	1.36	0.67–2.77	0.40	0.31	1.38	0.43—4.39	0.58	0.32
Mechanical compression	1.81	0.79–4.15	0.16	0.59	3.07	1.05 – 8.90	0.04	1.12
Cons	-	-	-		2.74	0.26–28.79	0.40	

Multivariable logistic model for ED ROSC, we tested goodness-of-fit test by Hosmer–Lemeshow, number of groups = 10, *P*-value = 0.85.

Overall primary and secondary outcomes are presented in Table 3. In the COVID-19 period, ED ROSC was statistically significantly lower than before the COVID-19 period; there were 51 total ED ROSC cases, of which 16 were in the COVID-19 period and 35 were before the COVID-19 period (26.67% vs. 46.43%, respectively, crude odd ratio (OR) 0.43 [95% confidence interval (CI) 0.21–0.88], *p* = 0.02), adjusted OR 0.21 [95% confidence interval (CI) 0.08–0.53], *p* < 0.001). For secondary outcomes, there were statistically significant differences in survival to admission between the COVID-19 period and before the COVID-19 period (25.00% vs. 40.79%, crude OR 0.48 [95% confidence interval (CI) 0.23–1.02], *p* = 0.06), adjusted OR 0.26 [95% CI 0.10–0.67], p = 0.005). However, the 30-day survival was 3.33% during the COVID-19 period and 10.53% before the COVID-19 period%, crude OR 0.29 [95% confidence interval (CI) 0.06–1.44], *p* = 0.13), adjusted OR 0.14 [95% CI 0.02–1.28], *p* = 0.08), The total number of good CPC outcomes was too small, crude OR 0.31 [95% confidence interval (CI) 0.03–2.80], *p* = 0.29), adjusted OR 0.24 [95% CI 0.01–4.10], *p* = 0.33).

**Table 3:** Primary and secondary outcomes of out-of-hospital cardiac arrest during the COVID-19 period and before the COVID-19 period

**Outcomes**	**During COVID-19 period** **(N = 60)**	**Before COVID-19 period** **(N = 76)**		**Univariable**		**Multivariable**	
			**variable**	**Odd ratio**^**a**^ **(95% CI)**	**p-value**	**Adjusted OR**^**c**^	***p*-value**
**Primary outcome**							
**ED ROSC**, N (%)	16 (26.67)	35 (46.05)	**covid/** **before covid period (base)**	^**a**^**0.43 (0.21–0.88)**	**0.02**	^**c**^**0.21 (0.08–0.53)**	** < 0.001**
**Secondary outcomes**							
**Survival to admission**, N (%)	15 (25.00)	31 (40.79)	**Covid /** **before covid period (base)**	^**a**^**0.48 (0.23–1.02)**	**0.06**	^**c**^**0.26 (0.10–0.67)**	**0.005**
**30-day survival**, N (%)	2 (3.33)	8 (10.53)	**Covid /** **before covid period (base)**	^**a**^**0.29 (0.06,1.44)**	**0.13**	^**c**^**0.14 (0.02–1.28)**	**0.08**
**30-day good CPC** (**Category 1–2)**, N (%)	1(1.67)	4(5.26)	**Covid /** **before covid period (base)**	^**a**^**0.31 (0.03–2.80)**	**0.29**	^**c**^**0.24 (0.01- 4.10)**	**0.33**

^a^ Crude odds ratio

^c^Adjusted odds ratio with age, comorbidities (diabetes, hypertension, dyslipidemia, chronic kidney disease, ischemic heart disease, cerebrovascular disease, airway disease), cardiac etiology, location of cardiac arrest, bystander CPR, EMS transport mode, initial rhythm and mode of compression

*EMS,* emergency medical service; *ROSC,* return of spontaneous circulation; *CPC,* cerebral performance category; *RD*, risk difference; *OR*, odds ratio; *CI*, confidence interval

In Table4, the hospital dispatch system subgroup analysis was performed specifically on patients from the hospital dispatch system, and differences were observed in the services between the two periods. There were 66 total cases; 33 during the COVID-19 period and 33 before the COVID-19 period. Baseline characteristics of the hospital dispatch system subgroup are presented in Table 4; the mean age was 63.23 years, and 71.21% were male. While mechanical chest compression was used more in the COVID-19 period than before (96.97% vs. 69.70%, *p* = 0.006), the ROSC before ED arrival was significantly lower during the COVID-19 period than during the prior period (15.15% vs. 42.42%, *p* = 0.03). Nonetheless, there were no statistically significant differences observed between the two groups in terms of etiology of cardiac arrest, location of occurrence of the OHCA, rate of bystander-witnessed events, rate of bystander CPR, EMS response time, patients with an initial shockable rhythm, defibrillation, endotracheal intubation, or prehospital CPR time.

**Table 4:** Baseline characteristics of out-of-hospital cardiac arrest During COVID-19 period and Before COVID-19 period (hospital dispatch system subgroup

Baseline characteristic	All (N = 66)	During COVID-19 period (N = 33)	Before COVID-19 period (N = 33)	p-value
Age, years (mean ± SD)	63.23 ± 19.34	61.21 ± 19.32	65.24 ± 19.45	0.40
Male sex, N (%)	47 (71.21)	24 (72.73)	23 (69.70)	1.00
Comorbidity status, N (%)	38 (57.58)	16 (48.48)	22 (66.67)	0.21
Hypertension	31 (46.97)	14 (42.42)	17 (51.52)	0.62
Diabetes mellitus	17 (25.76)	9 (27.27)	8 (24.24)	1.00
Dyslipidemia	17 (25.76)	7 (21.21)	10 (30.30)	0.57
Chronic kidney disease	7 (10.61)	3 (9.09)	4 (12.12)	1.00
Ischemic heart disease	10 (15.15)	5 (15.15)	5 (15.15)	1.000
Cerebrovascular disease	7 (10.61)	3 (9.09)	4 (12.12)	1.000
Asthma/COPD	3 (4.55)	0(0)	3 (9.09)	0.24
Cardiac etiology, N (%)	28 (42.42)	14 (42.42)	14 (42.42)	1.00
Public location, N (%)	21 (31.82)	12 (36.36)	9 (27.27)	0.60
Bystander-witnessed, N (%)	65 (98.48)	32 (96.97)	33 (100.00)	1.00
Bystander CPR, N (%)	53 (80.30)	28 (84.85)	25 (75.76)	0.54
EMS response, N (%)				
-Advanced life support (ALS)	63 (95.45)	32 (96.97)	31 (93.94)	1.00
-Basic life support (BLS)	3 (4.55)	1 (3.03)	2 (6.06)	1.00
EMS response time, minutes	11.87 ± 5.08 (*n* = 39)	12.33 ± 5.75 (*n* = 24)	11.13 ± 3.85 (*n* = 15)	0.48
Initial shockable rhythm, N (%)	19 (28.79)	10 (30.30)	9 (27.27)	1.00
Mode of compression, N (%)				
-Mechanical devices	55 (83.33)	32 (96.97)	23 (69.70)	0.006
-Manual	11 (16.67)	1 (3.03)	10 (30.30)	
Defibrillation, N (%)	22 (33.33)	15 (45.45)	7 (21.21)	0.07
Endotracheal intubation, N (%)	66 (100.00)	33 (100.00)	33 (100.00)	1.00
Prehospital CPR time, min	27.97 ± 13.64 (*n* = 59)	30.30 ± 13.78 (*n* = 30)	25.55 ± 13.29 (*n* = 29)	0.18
ROSC before ED arrival, N (%)	19 (28.79)	5 (15.15)	14 (42.42)	0.03

*CPR,* cardiopulmonary resuscitation; *ROSC* return of spontaneous circulation; *CPC* cerebral performance category

Patient cardiac arrest outcomes of the hospital dispatch system subgroup are presented in Table 5. We found no statistically significant differences between during COVID-19 period and the before-COVID-19 period: ED ROSC (adjusted OR 0.33 [95% CI 0.08–1.47], *p* = 0.15), survival to admission, (adjusted OR 0.36 [95% CI 0.07–1.84], *p* = 0.22), 30-day survival (adjusted risk different (RD)—0.01 [95% CI − 0.21 to 0.03], *p* = 0.14) and 30-day good CPC score (adjusted RD -0.04 [95% CI − 0.16 to 0.07], *p* = 0.45) respectively.

**Table 5:** Primary and secondary outcomes of out-of-hospital cardiac arrest between During COVID-19 period and Before COVID-19 period (hospital dispatch system subgroup)

**Outcomes**	**During COVID-19 period** **(*N* = 33)**	**Before COVID-19 period** **(*N* = 33)**		**Univariable**		**Multivariable**	
			**variable**	**Odd ratio**^**a**^ **or RD**^**b**^ **(95% CI)**	***p*-value**	**Adjusted OR**^**c**^** or Adjusted RD**^**d**^** (95% CI)**	***p*-value**
**Primary outcome**							
**ED ROSC**, N (%)	8 (24.24)	14 (42.42)	**covid/** **before covid period (base)**	^**a**^**0.43 (0.15–1.25)**	**0.12**	^**c**^**0.33 (0.08–1.47)**	**0.15**
**Secondary outcomes**							
**Survival to admission**, N (%)	7 (21.21)	11 (33.33)	**Covid /** **before covid period (base)**	^**a**^**0.54** **(0.18–1.63)**	**0.27**	^**c**^**0.36 (0.07–1.84)**	**0.22**
**30-day survival**, N (%)	0 (0)	4 (12.12)	**Covid /** **before covid period (base)**	^**b**^**-0.12 (-0.23, -0.01)**	**0.03**	^**d**^**-0.09 (-0.21, 0.03)**	**0.14**
**30-day good CPC** (**Category 1–2)**, N (%)	0 (0)	2 (6.06)	**Covid /** **before covid period (base)**	^**b**^**-0.06 (-0.14, 0.21)**	**0.15**	^**d**^**-0.04 (- 0.16, 0.07)**	**0.45**

^a^ Crude odds ratio

^b^ Crude risk different (used risk different due to zero outcome)

^c^Adjusted odds ratio with age, comorbidities (diabetes, hypertension, dyslipidemia, chronic kidney disease, ischemic heart disease, cerebrovascular disease, airway disease), cardiac etiology, location of cardiac arrest, bystander CPR, EMS transport mode, initial rhythm, and mode of compression

^d^Adjusted risk difference with age, comorbidities (diabetes, hypertension, dyslipidemia, chronic kidney disease, ischemic heart disease, cerebrovascular disease, airway disease), cardiac etiology, location of cardiac arrest, bystander CPR, EMS transport mode, initial rhythm, and mode of compression

*CPC,* cerebral performance category; *ROSC,* return of spontaneous circulation; *OR*, odds ratio; *CI*, confidence interval; *RD*, risk difference

## Discussion

In this study, we compared the characteristics and outcomes of OHCA patients before and during the COVID-19 pandemic in Thailand. The main findings were similar to other studies in developed countries. The number of ED ROSC in OHCA patients was significantly lower during the COVID-19 period than before the COVID-19 period, congruent with several previous studies, particularly in the United States (US) (27,36–38). This could be the consequence of the Thai prehospital management that was adopted from the US, the Anglo-American model, which gave cares based in the ED, and EMS follows the philosophy of “scoops and runs,” the similarities in outcomes of ED ROSC would be expected (38).

Moreover, the proportion of OHCA patients who survived to admit was significantly lower during the COVID-19 period than in the comparison period, similar to previous reports from several studies (16,24,25,38–42). The low numbers of ED ROSC and survived until hospital admission during the COVID-19 period were multifactorial. Yet, they are presumably due to stay-at-home directives, fear of disease transmission during hospital access, and the delayed first medical contact from the overloading of public emergency numbers, particular in the EMS group, that slowed the initial healthcare-seeking behaviors, and the unrecognized risks and symptoms of severe diseases at home by patients and relatives. Nevertheless, our study did not show statistical differences in 30-day survival and a good 30-day CPC score between the COVID-19 and preceding periods.

The suboptimal outcomes during pandemics are presumably affected by the overwhelming of health care resources, especially in countries where the pandemic has magnified inequities in the healthcare accessibility (44). Moreover, the severity of the outcomes in excess OHCA and death could be influenced by uncontrolled comorbidities stemming from health service inaccessibility and COVID-19–imposed infections and pandemic-related environmental, emotional, and economic stressors (27). Furthermore, the modification of cardiac arrest guidelines to indicate delayed intubation, covering patients’ mouth and nose before chest compression, and complete personal protective suits delayed initiation of CPR and consequently affected the irreversible outcomes (18).

The significant differences in baseline characteristics between the two groups were witnessed cardiac arrest and mode of chest compression. A previous study reported a significant association between OHCA during the COVID-19 period and comorbidities, notably diabetes and hypertension (31). Similarly, our research found a concomitant in the overall prevalence of comorbidities (including hypertension, diabetes mellitus, dyslipidemia, chronic kidney disease, ischemic heart disease, cerebrovascular disease, and asthma/COPD) between the two periods. When compared with the meta-analysis (37), the OHCA patients’ characteristics in the COVID-19 period had the same high proportion of cardiac arrest at home and low proportion of endotracheal intubation during prehospital care. The significant increase in the use of mechanical chest compression shown in our finding was presumably from the recommendation of the World Health Organization and the AHA to use mechanical chest compression instead of manual compression to minimize the risk of COVID-19 transmission to health care providers and minimize personal contact to a patient during CPR, particularly in COVID-19 suspected patients or patients who had no serology test (18). In contrast, studies in Italy showed no difference in the use of mechanical CPR between the two periods (16,39). Additionally, the proportion of bystander CPR in our study was low and this was similar to previous studies in both developed and developing countries which indicated a need for community education on CPR and chest compression (37). Nevertheless, our study needs a more extensive data collection to identify the factors associated with and causal relations to the decrease of ED ROSC and survival to admission during the COVID-19 pandemic.

A subgroup analysis of only the EMS arrival cases (Table 4) in this study found that the average EMS response time during the COVID-19 period was approximately 1 min longer than before the COVID-19 period (without a statistically significant difference), which could imply that the time lag for universal precaution preparation did not affect the overall EMS response time. However, because the numbers of EMS cases in the study were small, a comparison in a larger sample size should be conducted to reach a more precise conclusion. In addition, the results affirmed the previous evidence about the correlation between ED ROSC and EMS response time, specifically that the shorter the EMS response time, the higher the proportion of ED ROSC cases (45). However, our results showed no significant differences in the ED ROSC and survival to admission in the hospital dispatch subgroup between the two groups, which could be limited by the study’s small sample size and lack of complete prehospital information.

## Limitations

There are some limitations to this study that applied to both study periods. First, this was a retrospective single-center study; we had under power (68%) when calculated back to detect a ED ROSC, which resulted in a small sample size as well as some gaps in data, notably response time and resuscitative intervention because at the time of operation there was no standard CPR record form. EMS personnel transporting OHCA patients to the hospital were from various area hospitals. Second, Bangkok has a two-level dispatch system; because dispatches to the hospitals come from the central dispatch center, there is an unspecified time between the initial emergency calls and the actual dispatch time that the hospital units have limited to collect. However, this time delay was typically minimal and likely had only minor effects on the overall time to treatment. Third, because this was a single-center study, the results were constrained in certain ways, and might not represent the services in other areas. However, the results showed a trend of prehospital care for OHCA in a developing country that had similar patient outcomes compared to other studies. Finally, there was a limitation in the COVID-19 period about cardiac arrest etiology because postmortem testing to confirm COVID-19 was not performed. We could not precisely guarantee the direct impact of the infection on OHCA outcomes (46). Nonetheless, the countries followed the common COVID-19 recommendations. Thus, the data might be interpreted as patient outcomes and prehospital care trends.

## Conclusion

During the COVID-19 pandemic in Thailand, ED ROSC and survival to admission in out-of-hospital cardiac arrest patients have been significantly decreased. Additionally, the pandemic’s Witness and mode of chest compression have been altered considerably, highlighting the significance of prehospital interventions and public health education initiatives. However, the factors' associations with ED ROSC and survival to admission outcomes during the covid 19 period require further investigation.

## Data availability statement

The data supporting this study’s findings are openly available in [Harvard Dataverse] at https://doi.org/10.7910/DVN/2W1RWC.

## Supplementary Information

Additional file1 Supplement1 Univariable and Multivariable analysis factors of Survival to admission of cases during COVID-19 period and before COVID-19 period Supplement 2 Univariable and Multivariable analysis factors of **30-day survival **of cases during COVID-19 period and before COVID-19 period Supplement 3 Univariable and Multivariable analysis factor of **30-day good CPC score** between During COVID-19 period and Before COVID-19 period
