# Supplementary material for: Characteristics and outcomes of out-of-hospital cardiac arrest patients before and during the COVID-19 pandemic in Thailand
Source: Int J Emerg Med. 2022 Sep 9;15:46. doi: 10.1186/s12245-022-00444-2 (PMC9461095; doi:10.1186/s12245-022-00444-2)
Supplement: Supplementary file 1 — Additional file1 Supplement1 Univariable and Multivariable analysis factors of Survival to admission of cases during COVID-19 period and before COVID-19 period Supplement 2 Univariable and Multivariable analysis factors of 30-day survival of cases during COVID-19 period and before COVID-19 period Supplement 3 Univariable and Multivariable analysis factor of 30-day good CPC score between During COVID-19 period and Before COVID-19 period [file 12245_2022_444_MOESM1_ESM.docx]

Supplement1

Univariable and Multivariable analysis factors of Survival to admission of cases during COVID-19 period and before COVID-19 period

| **Variable** | **Univariable** | | | | **Multivariable** | | | |
| --- | --- | --- | --- | --- | --- | --- | --- | --- |
|  | **Odd ratio** | **95%CI** | **P value** | **coef** | **Odd ratio** | **95%CI** | **P value** | **coef** |
| Covid period | 0.48 | 0.23-1.02 | 0.06 |  | 0.26 | 1.10 -0.67 | 0.005 | -1.34 |
| age | 0.99 | 0.97-1.01 | 0.19 |  | 0.97 | 0.95 - 0.99 | 0.05 | -0.03 |
| DM | 1.59 | 0.72-3.49 | 0.25 |  | 2.06 | 0.68 - 6.23 | 0.20 | 0.72 |
| HT | 0.80 | 0.39-1.63 | 0.55 |  | 0.70 | 0.23 - 2.15 | 0.53 | -0.36 |
| Dyslipidemia | 1.44 | 0.65-3.19 | 0.37 |  | 1.80 | 0.51 - 6.30 | 0.36 | 0.59 |
| Chronic kidney disease | 1.17 | 0.43-3.20 | 0.76 |  | 0.94 | 0.26 - 3.38 | 0.92 | -0.07 |
| Heart disease | 0.81 | 0.29-2.28 | 0.70 |  | 0.71 | 0.21 - 2.33 | 0.57 | -0.35 |
| Cerebrovascular disease | 1.17 | 0.43-3.20 | 0.76 |  | 1.66 | 0.51 - 5.39 | 0.40 | 0.51 |
| Chronic lung disease | 2.62 | 0.67-10.28 | 0.17 |  | 1.80 | 0.37 - 8.69 | 0.46 | 0.59 |
| Cardiac etiology | 0.67 | 0.31-1.48 | 0.32 |  | 0.77 | 0.29 - 2.09 | 0.61 | -0.26 |
| Public location | 1.78 | 0.87-3.65 | 0.12 |  | 1.28 | 0.54 - 2.99 | 0.58 | 0.24 |
| Bystander CPR | 0.96 | 0.47-1.99 | 0.92 |  | 10.85 | 1.17 - 100.66 | 0.04 | 2.38 |
| EMS transport | 0.56 | 0.27-1.16 | 0.12 |  | 0.08 | 0.01 – 0.86 | 0.04 | -2.47 |
| Initial shockable rhythm | 1.81 | 0.86-3.80 | 0.12 |  | 1.88 | 0.57 - 6.19 | 0.30 | 0.63 |
| Mechanical compression | 1.76 | 0.75-4.15 | 0.20 |  | 3.10 | 1.03 – 9.32 | 0.04 | 1.13 |
| cons | - | - | - |  | 1.46 | 0.13-16.05 | 0.76 | 0.38 |

Supplement 2

Univariable and Multivariable analysis factors of **30-day survival** of cases during COVID-19 period and before COVID-19 period

| **Variable** | **Univariable** | | | | **Multivariable** | | | |
| --- | --- | --- | --- | --- | --- | --- | --- | --- |
|  | **Odd ratio** | **95%CI** | **P value** | **coef** | **Odd ratio** | **95%CI** | **P value** | **coef** |
| Covid period | 0.29 | 0.06-1.44 | 0.13 | -1.23 | 0.14 | 0.02 -1.28 | 0.08 | -1.95 |
| age | 0.99 | 0.96-1.02 | 0.56 | -0.01 | 0.96 | 0.90 - 1.02 | 0.19 | -0.04 |
| DM | 1.96 | 0.52-7.38 | 0.32 | 0.67 | 0.78 | 0.09 - 6.84 | 0.82 | -2.48 |
| HT | 4.54 | 0.92- 22.24 | 0.06 | 1.51 | 5.04 | 0.41 - 62.47 | 0.21 | 1.62 |
| Dyslipidemia | 3.20 | 0.87-11.81 | 0.08 | 1.16 | 0.85 | 0.70 - 10.27 | 0.90 | -0.16 |
| Chronic kidney disease | 1.60 | 0.31-8.19 | 0.57 | 0.47 | 1.07 | 0.09 - 12.68 | 0.96 | 0.06 |
| Heart disease | 1.50 | 0.29-7.64 | 0.62 | 0.41 | 0.54 | 0.04 - 6.85 | 0.64 | -0.61 |
| Cerebrovascular disease | 2.95 | 0.69-12.57 | 1.14 | 1.08 | 9.63 | 0.74 - 125.62 | 0.08 | 2.26 |
| Chronic lung disease | 8.57 | 1.76-41.68 | 0.008 | 2.15 | 10.09 | 0.86 - 117.99 | 0.07 | 2.31 |
| Cardiac etiology | 2.32 | 0.63-8.47 | 0.20 | 0.84 | 17.21 | 0.83 - 356.61 | 0.07 | 2.84 |
| Public location | 11.62 | 1.43-94.48 | 0.02 | 2.45 | 15.57 | 0.74 - 328.36 | 0.08 | 2.75 |
| bystander | 0.63 | 0.16-2.55 | 0.52 | -0.46 | 0.32 | 0.01 - 10.26 | 0.52 | 2.38 |
| EMS transport | 0.68 | 0.19-2.56 | 0.58 | -0.37 | 2.98 | 0.12 – 74.23 | 0.51 | -1.15 |
| Initial shockable rhythm | 3.10 | 0.63-15.18 | 0.16 | 1.13 | 18.60 | 0.56 - 616.87 | 0.10 | 2.92 |
| Mechanical compression | 1.48 | 0.30-7.31 | 0.63 | 0.39 | 20.25 | 0.78 – 526.19 | 0.07 | 3.01 |
| cons | - | - | - |  | 0.000 | 4.33e-8 -0.86 | 0.05 | -8.55 |

Supplement 3

Univariable and Multivariable analysis factor of **30-day good CPC score** between During COVID-19 period and Before COVID-19 period

| **Variable** | **Univariable** | | | | **Multivariable** | | | |
| --- | --- | --- | --- | --- | --- | --- | --- | --- |
|  | **Odd ratio** | **95%CI** | **P value** | **coef** | **Odd ratio** | **95%CI** | **P value** | **coef** |
| Covid period | 0.31 | 0.03-2.80 | 0.29 | -1.19 | 0.24 | 0.01 -4.10 | 0.33 | -1.41 |
| age | 0.99 | 0.95-1.04 | 0.71 | -0.01 | 0.98 | 0.90 - 1.06 | 0.61 | -0.02 |
| DM | 0.69 | 0.07-6.35 | 0.74 | -0.38 | 0.38 | 0.01 -10.25 | 0.56 | -0.98 |
| HT | 1.57 | 0.25-9.71 | 0.63 | 0.45 | 0.37 | 0.01 - 13.40 | 0.58 | -1.01 |
| Dyslipidemia | 1.98 | 0.32-12.37 | 0.47 | 0.68 | 5.21 | 0.08 - 347.90 | 0.44 | 1.65 |
| Chronic kidney disease | 1 (omit) | - | - | 0 | 1 (omit) | - | - | - |
| Heart disease | 1.47 | 0.16-13.91 | 0.74 | 0.39 | 0.56 | 0.01 - 32.23 | 0.78 | -0.57 |
| Cerebrovascular disease | 1.57 | 0.17-14.85 | 0.70 | 0.45 | 3.03 | 0.11 - 84.55 | 0.51 | 1.11 |
| Chronic lung disease | 11.81 | 1.69-82.55 | 0.01 | 2.47 | 18.03 | 0.85 - 381.81 | 0.06 | 2.89 |
| Cardiac etiology | 3.41 | 0.55-21.22 | 0.19 | 1.23 | 18.49 | 0.31 - 1113.25 | 0.16 | 2.92 |
| Public location | 4.73 | 0.52-43.50 | 0.17 | 1.56 | 2.77 | 0.18 - 43.25 | 0.47 | 1.02 |
| bystander | 1.01 | 0.16-6.27 | 0.99 | 0.01 | 3.11 | 0.01 - 971.80 | 0.70 | 1.13 |
| EMS transport | 0.70 | 0.11-4.31 | 0.70 | -0.36 | 0.16 | 0.00 – 59.86 | 0.54 | -1.85 |
| Initial shockable rhythm | 1.09 | 0.18-6.72 | 0.93 | 0.08 | 4.29 | 0.05 - 349.61 | 0.52 | 1.46 |
| Mechanical compression | 1.46 | 0.16-13.50 | 0.74 | 0.38 | 8.08 | 0.10 – 642.60 | 0.35 | 2.09 |
| cons | - | - | - | - | 0.00 | 6.88e-8 -90.08 | 0.26 | -6.00 |
